# Orf116b Induces Pollen Abortion in a Novel Cotton (*Gossypium hirsutum* L.) Cytoplasmic Male Sterile Line J4A

**DOI:** 10.3390/ijms252212257

**Published:** 2024-11-14

**Authors:** Min Li, Aziz Khan, Jie Zheng, Jingyi You, Li Chen, Najeeb Ullah, Songguo Wu, Xiaoshuang Wei, Munsif Ali Shad, Ruiyang Zhou, Lingqiang Wang

**Affiliations:** 1 State Key Laboratory for Conservation and Utilization of Subtropical Agro-Bioresources, College of Agriculture, Guangxi University, Nanning 530004, China; limingas2023@163.com (M.L.);; 2 National Key Laboratory of Non-Food Biomass and Enzyme Technology, National Engineering Research Center for Non-Food Biorefinery, Institute of Grand Health, Guangxi Academy of Sciences, Nanning 530007, China; 3 National Key Laboratory of Cotton Bio-Breeding and Integrated Utilization, Institute of Cotton Research, Chinese Academy of Agricultural Sciences (ICR, CAAS), Anyang 455000, China; 4 Agriculture Research Station, Office of VP for Research and Graduate Studies, Qatar University, Doha 2713, Qatar

**Keywords:** mitochondrial genome, J4A, membrane-based yeast two-hybrid, mitochondrial gene, pollen abortion

## Abstract

Cytoplasmic male sterile (CMS) systems have great potential in hybrid seed production in many plants. However, the incompatibility between the cytoplasmic and nuclear genes and the availability of fewer CMS lines limit the system’s application in cotton heterosis. Therefore, the present study sequenced the mitochondrial (mt) genomes of a novel cotton (*Gossypium hirsutum* L.) CMS line J4A and its cytoplasmic nuclear homologous maintainer line J4B to investigate the mechanism underlying CMS and improve its application. A novel CMS gene, *orf116b*, was identified in the CMS line. Ectopic expression of *orf116b* in *Escherichia coli* suppressed growth, while its overexpression in Arabidopsis, rice, tobacco, and cotton led to complete or partial male sterility. Further analysis of anthers revealed mt disruption in J4A with higher levels of hydrogen peroxide (H_2_O_2_) and malondialdehyde (MDA), but lower levels of ATP and ribosomal protein (RP) than in J4B. Finally, a membrane-based yeast two-hybrid (MYTH) assay and bimolecular fluorescence complementation (BiFC) assays demonstrated that orf116b probably interacts with an anther-specific protein, LAT52. These observations collectively proved that orf116b is associated with early and stable pollen abortion in cotton, providing a foundation for further research on cotton fertility restoration and heterosis breeding.

## 1. Introduction

Cytoplasmic male sterility (CMS), a condition marked by maternal inheritance and characterized by nonfunctional pollen, is primarily caused by chimeric genes in the mitochondrial (mt) genome. This condition can be suppressed or counteracted by nuclear genes known as the restorer of fertility (Rf) genes [1,2], most of which encode pentatricopeptide repeat proteins and regulate CMS largely at the post-transcriptional level [3,4,5,6,7]. CMS lines have been widely exploited for hybrid seed production in diverse plant species [8]. However, due to the limited availability of CMS germplasm, less attention has been given to its application in cotton (*Gossypium hirsutum* L.), a plant that produces a renewable and biodegradable natural fiber with a significant role in the economy, society, culture, ecology, and technology [9]. The current cotton breeding programs primarily employ the CMS-D2 line, which contains the cytoplasm of the diploid wild species *Gossypium harknessii* Brandegee (D2) [10,11]. Research has proven that in the CMS-D2 line ZBA, the mt protein orf610a interacts with the nuclear protein RD22 to initiate pollen abortion [11]. Nevertheless, the popularization of CMS-D2 has been challenging due to the incompatibility between the mt and nuclear genomes and the restricted availability of restorer lines [12]. In such a scenario, creating a new CMS/Rf system would benefit hybrid seed production. Furthermore, the CMS/Rf system would be an excellent model for studying mt-nuclear interactions.

The molecular mechanisms underlying CMS have been elucidated in many species, such as maize [13,14], rice [6,15,16], rapeseed [17,18], and wheat [19,20]. Based on the reports in these species, four CMS models have been proposed, namely, the energy metabolism disorder (*orf279* of wheat T-CMS and *PRESATP6* of sugar beet Owen CMS) [21,22,23], cytotoxicity (*orf346* of *Brassica napus* Nsa-CMS and *urf13* of maize T-CMS) [17,23], abnormal programmed cell death (PCD) (peach) [24], and retrograde regulation (*orf355* of corn S-CMS) [14,25]. These reports indicated that the mechanisms underlying CMS vary across species and cultivars. Therefore, it is critical to understand the specific mechanism underlying CMS in cotton to exploit it in heterosis and hybrid crop breeding.

Therefore, the present study aimed to elucidate the mechanism inducing CMS in a novel cotton line, J4A. We sequenced the entire mt genomes of the CMS line J4A (mutant type) and its cytoplasmic nuclear homologous maintainer line J4B (wild type) using Illumina Hi-seq and PacBio technologies. Then, their mt genomes were compared to assess the differences in DNA organization and identify a CMS gene. The identified CMS gene (*orf116b*) was overexpressed in both prokaryotic and eukaryotic models to determine its effects on mt function or/and CMS. Further, a yeast two-hybrid (Y2H) cotton anther-related DUAL membrane library was screened, and a bimolecular fluorescence complementation (BiFC) assay was performed to identify a protein interacting (LAT52, an anther-specific protein) with the CMS protein (orf116b) and elucidate the sterility-associated nuclear–cytoplasmic interaction. The study’s findings will provide useful information for restoring fertility in J4A and facilitating hybrid breeding and seed production in cotton.

## 2. Results

### 2.1. Abnormal Proliferation of Tapetal Cells in J4A

**Figure 1:**
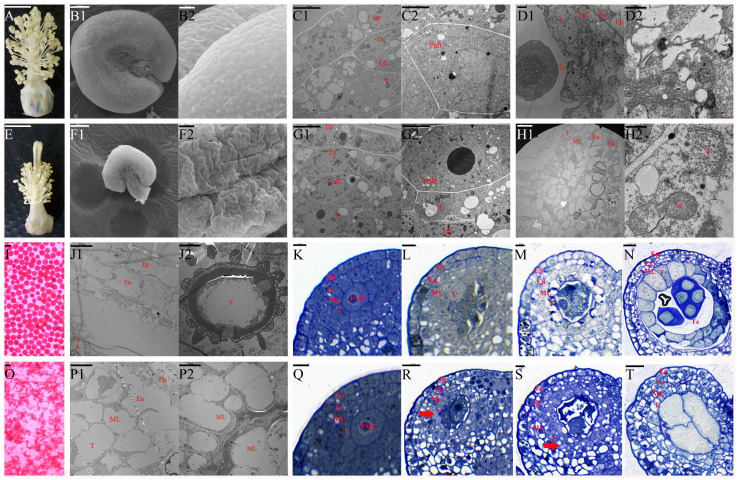
Morphological and cytological features of J4B and J4A cotton anthers. Morphology of the filament morphology of J4B (**A**) and J4A (**E**). SEM images of the frontal view of mature pollen grains in anthers of J4B (**B1**) and J4A (**F1**); SEM images of the lateral view of mature pollen grains in anthers of J4B (**B2**) and J4A (**F2**). TEM images of J4B and J4A anthers at the pollen mother cell stage (**C1**,**C2**,**G1**,**G2**), the tetrad stage (**D1**,**D2**,**H1**,**H2**), and the mature pollen grain stage (**J1**,**J2**,**P1**,**P2**). I_2_-KI staining of J4B and J4A pollen grains (**I**,**O**). Semi-thin sections of J4B and J4A anthers during the pollen mother cell stage (**K**,**Q**), between the pollen mother cell stage and meiosis stage (**L**,**R**), at the meiosis stage (**M**,**S**), and at the tetrad stage (**N**,**T**). Ep, exodermis; En, endoderm; ML, middle-level; T, tapetum; N, nucleus; PMC, pollen mother cell; M, mitochondrion; Te, tetrad; P, pollen. Scale bars of (**A**) and (**E**) correspond to 1 cm; scale bars of (**B1**,**D2**,**F1**,**H2**) correspond to 100 μm; scale bars of (**B2**,**F2**,**N**,**R**,**T**) correspond to 20 μm; scale bars of (**C1**,**C2**,**D1**,**G1**) correspond to 5 μm; scale bars of (**G2**,**I**,**O**) correspond to 50 nm; scale bars of (**H1**,**J1**,**J2**,**K**–**M**,**P1**,**P2**,**Q**,**S**) correspond to 10 μm.

Previously, we developed J4A, a CMS mutant line of cotton. The present study assessed the abortion type and explored the morphological and cytological features of the anthers of J4A and its maintainer line J4B using scanning electron microscope (SEM) and transmission electron microscopic (TEM) analyses. Compared to J4B, J4A had smaller anthers that appeared to be shrunk without any visible cracks, but with a shorter filament at flowering (Figure 1A,B1,B2,E,F1,F2). The iodine–potassium iodide (I_2_-KI) staining proved the absence of pollen in J4A anthers at flowering (Figure 1I,O), indicating pollen abortion. In both lines, the pollen mother cells were at the center of a four-layered sac (epidermis, endothecium, middle layer, and tapetum) (Figure 1C1,C2,G1,G2). In J4B, the pollen sac had a tetrad structure at the tetrad stage (Figure 1D1), with degraded tapetal cells and middle layer cells (Figure 1D2). At the mature pollen grain stage, J4B tapetal cells were completely degraded, and the epidermis and endothecium cells were vacuolated (Figure 1J1); moreover, mature pollen grains with spikes were visible in the pollen sac cavity (Figure 1J2). In J4A, the pollen sac had no tetrad structure at the tetrad stage (Figure 1H1). Here, the pollen sac had four layers of deformed cells; however, the tapetal cells were not degraded. In the tapetal cells, the mt membrane was deformed and sometimes even ruptured (Figure 1H2), indicating structural damage to J4A mt. At the mature pollen grain stage, the J4A anthers had no pollen grains (Figure 1P2), consistent with the results based on I_2_-KI staining (Figure 1O). Here, the exoderm, endothelium, middle layer, and tapetum were vacuolated and exhibited disordered arrangements (Figure 1P1). In addition, the pollen sac cavity appeared deformed and contracted, displaying a thick solid line-like appearance (Figure 1P2). These observations indicated that anther abortion in J4A occurred between the pollen mother cell and tetrad stages.

Furthermore, semi-thin sections of anthers at four stages (pollen mother cell stage, late stage of pollen mother cell, meiosis stage, and tetrad stage) were analyzed to confirm the stage of J4A abortion. This approach revealed that the J4A anthers developed normally during the pollen mother cell stage (Figure 1K,Q), consistent with the TEM observation. However, from the pollen mother cell stage, the tapetal cells of the J4A anthers did not undergo normal programmed cell enlargement like those of the fertile line J4B, but began to proliferate abnormally (Figure 1L,R). Subsequently, during the meiosis stage, the tapetal layer of the sterile line J4A continued to proliferate abnormally and formed small and disordered multilayered cells (Figure 1S), while those of the fertile line J4B expanded and began to undergo vacuolization (Figure 1M). At the same time, the pollen mother cells of J4A began to undergo vacuolization. Further, during the tetrad stage, the tapetal cells that underwent vacuolization started undergoing programmed degradation in J4B (Figure 1N), forming the microspore structures. However, there was no microspore structure in the J4A pollen sac cavity (Figure 1T), and the original pollen mother cells completely degraded and formed a cavity. The tapetal cells did not degrade, and the entire pollen sac appeared severely wrinkled and deformed by the mature pollen grain stage. These results confirmed that anther abortion in J4A occurred from the late pollen mother cell stage.

### 2.2. The mt Genome Organization and Sequences in J4A and J4B

**Figure 2:**
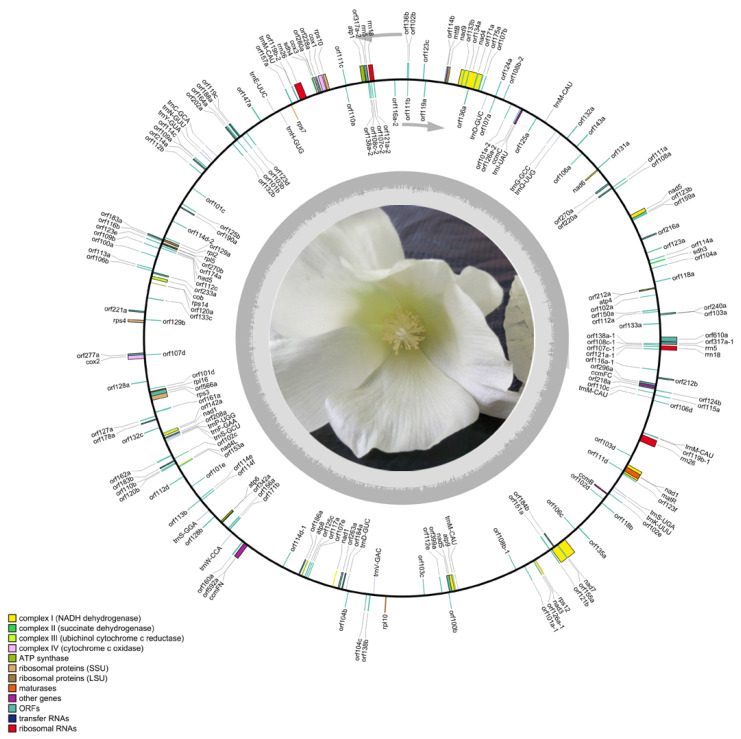
Circular map of the mt genome of the cotton CMS line J4A. The outer circle represents the mt genes. The details of the DNA strands transcribed clockwise (+) and counterclockwise (−) are displayed inside and outside the circles, respectively. The color near each gene indicates the function, as shown in the legend on the left side.

Further, to explore CMS-associated genes, the complete mt genomes of J4A and J4B were sequenced and assembled using the Illumina NovaSeq and PacBio Sequel technologies (Figure 2, Table S1). Illumina sequencing generated 11443.8 Mb and 8454.8 Mb raw data (Table S2) and 10991.4 Mb and 8147.3 Mb clean data for J4A and J4B, respectively, with Q20 values of 95.22% and 95.37%, Q30 values of 86.55% and 86.95%, and GC content of 39.42% and 38.99%, respectively. PacBio sequencing generated 460,703,329 bp and 574,874,134 bp of total data and 92,527 and 11,7674 filtered subreads for J4A and J4B, respectively (Table S3), with a maximum of 209,202 bp and 194,861 bp subreads. After mixing and assembling the PacBio and Illumina sequences, we obtained 634,005 bp (Figure 2) and 677,303 bp (Figure S1) long mtDNA for J4A and J4B, respectively, with GC contents of 44.91% and 44.96%. No unknown bases (N) were detected in the assembled sequences of these two lines, confirming the suitability of the mt genomes for subsequent comparative analysis.

**Figure 3:**
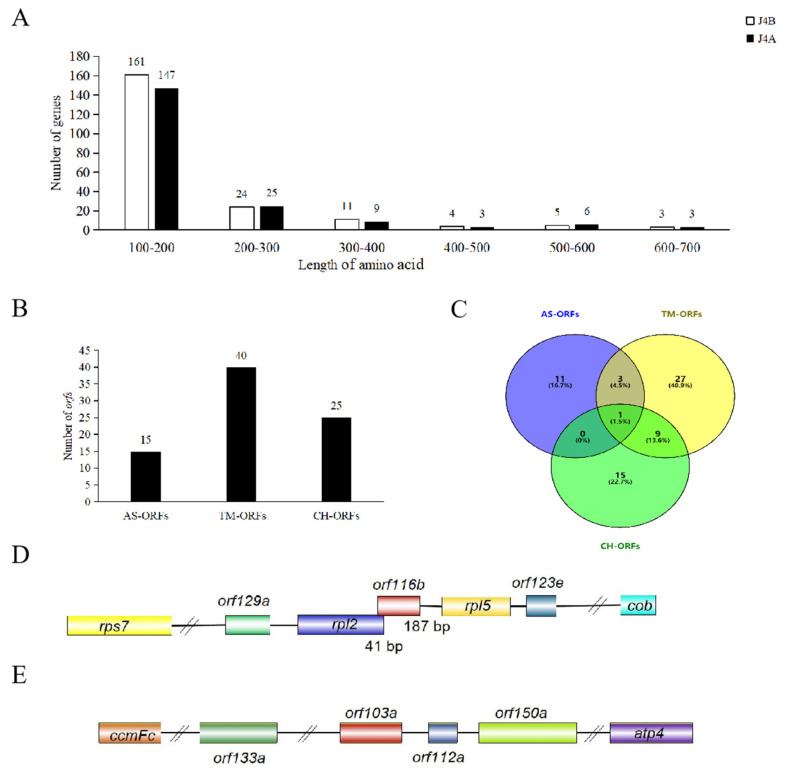
Comparative analysis of the J4A and J4B genomes and mining of the ORFs. (**A**) Distribution of mt genes encoding proteins with different numbers of amino acids in J4B and J4A. (**B**) Number of AS-ORFs, TM-ORFs, and CH-ORFs in the J4A mt genome. (**C**) Venn diagram shows the relationship among AS-ORFs, TM-ORFs, and CH-ORFs. (**D**) Structure of *orf116b* in the J4A mt genome. (**E**) Structure of *orf103a* in the J4A mt genome. AS-ORFs, J4A-specific ORFs; TM-ORFs, ORFs with transmembrane domains; CH-ORFs, chimeric ORFs. Boxes represent coding sequences, different colors represent different coding genes, and horizontal lines represent the flanking regions.

Furthermore, in the mt genomes of J4A and J4B, we found 193 and 208 annotated genes (encoding proteins with more than 100 amino acids), respectively, including ORFs (128 in J4A and 141 in J4B), mt protein-coding genes (36 in both lines), transfer RNAs (tRNA; 23 in J4A and 26 in J4B), and ribosomal RNAs (rRNA; 6 in J4A and 5 in J4B) (Tables S4 and S5). Among the 36 mt protein-coding genes, 3 in J4B (*nad4* and *nad9* encoding NADH ubiquinone oxidoreductase and *mttB* encoding transporters) had multiple copies (Table S4), while all in J4A had a single copy. In both lines, eight genes had introns, of which four (*nad1*, *nad2*, *nad5*, and *nad7*) had four introns each and another four (*cox2*, *ccmFC*, *rps3*, and *rps10*) had one intron each. The length of these mt protein-coding genes ranged from 303 bp (*nad4L*) to 2013 bp (*nad5*). The start codon of most genes was ATG, while that of *nad1*, *nad4L*, and *rps10* was ACG, and that of *mttB* was ATT. The mt genomes of both J4A and J4B had seven ribosomal protein small (RPS) subunit-encoding genes (*rps3*, *rps4*, *rps7*, *rps10*, *rps12*, *rps14*, and *rps19*) and four ribosomal protein large (RPL) subunit-encoding genes (*rpl10*, *rpl16*, *rpl2*, and *rpl5*). Subsequent annotation of the genes using five open databases showed that NR and COG/KOG accounted for the maximum annotated genes, whereas GO and KEGG accounted for the fewest (Figure S2). Among all the mt genes, 16 of J4B and 15 of J4A were annotated in all five databases. These mt genes were grouped into six classes based on the number of amino acids in the encoded proteins (Figure 3A; 100–200, 200–300, 300–400, 400–500, 500–600, and 600–700 amino acids), of which approximately 76% in both J4A and J4B encoded proteins with 100–200 amino acids.

### 2.3. A Novel Male-Sterile Gene orf116b in the J4A mt Genome Co-Transcribes with the Neighboring Genes rpl2 and rpl5

**Figure 4:**
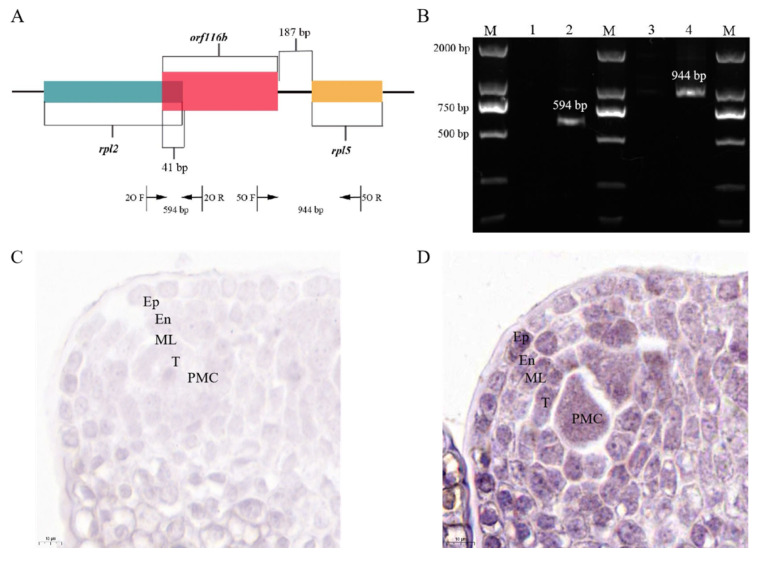
Co-transcription and in situ hybridization analyses of *orf116b*. (**A**) Gene structure of *orf116b* in J4A. The figure shows the template region which the primers target. (**B**) PCR validation of *orf116b* co-transcription with the upstream *rpl2* gene and the downstream *rpl5*. M, Maker 2000; Lane 1, J4B anther cDNA as template and 2O F/R primers (no co-transcription between *orf116b* and *rpl2* in J4B); Lane 2, J4A anther cDNA as template and 2O F/R primers (co-transcription of *orf116b* and *rpl2* in J4A); Lane 3, J4B anther cDNA as template and 5O F/R primers; Lane 4, J4A anther cDNA as template and 5O F/R primers. In situ hybridization of *orf116b* on the cross-sections of J4B (control) (**C**) and J4A (positive) (**D**) Anthers at pollen mother cell stage. The analysis proves that orf116b is expressed in J4A. Ep, exodermis; En, endoderm; ML, middle-level; T, tapetum; PMC, pollen mother cell.

We found 15 ORFs specific to the CMS line (AS-ORFs), 40 ORFs with transmembrane domains (TM-ORFs), and 25 chimeric ORFs (CH-ORFs) in the mt genome of J4A compared with J4B (Figure 3B,C, Table S5). Among these, the *orf116b* gene was uniquely found in the CMS line J4A, and its predicted protein was characterized by two transmembrane segments (Figure S3A). Subsequent sequence analysis showed that *orf116b* was located 187 bp (non-coding region) upstream of *rpl5* (Figure 3D), and its 3′ end overlapped with a 41 bp stretch at the 5′ end of *rpl2*. Although *orf103a* is specifically present in the J4A mt genome, and its predicted protein is characterized by the presence of one transmembrane domain (Figure 3E and Figure S3B), it is located at a considerable distance from the conserved mt genes. Further, to assess whether *orf116b* co-transcribed with their adjacent conserved genes *rpl2* and *rpl5*, a reverse transcription PCR (RT-PCR) was performed using the cDNA obtained from J4A and J4B anthers, 2O F/R (spanning *orf116b* and its adjacent protein-coding genes *rpl2*), and 5O F/R (spanning *orf116b* and its adjacent protein-coding genes *rpl5*) primers (Figure 4A). No target band was detected after PCR using the J4B anther cDNA and the 2O F/R primers (Figure 4B, Lane 1), indicating no co-transcription between *orf116b* and *rpl2* in J4B. Similarly, no co-transcription was found between *orf116b* and *rpl5* in J4B (5O F/R; Figure 4B, Lane 3). Meanwhile, an amplicon was detected after PCR with J4A anther cDNA and 2O F/R primers (Figure 4B, Lane 2), indicating co-transcription of *orf116b* and *rpl2* in J4A. A similar reaction with the J4A anther cDNA and 5O F/R primers revealed *orf116b* and *rpl5* co-transcription in J4A (Figure 4B, Lane 4). These results confirmed that *orf116b* co-transcribed with the neighboring genes *rpl2* and *rpl5* in the CMS line J4A.

Subsequently, to verify the expression of *orf116b* in the tapetal cells, in situ hybridization (CISH; BCIP/NBT color) was carried out in J4B and J4A anthers from the pollen mother cell stage before the occurrence of anther abortion. The expression of *orf116b* was not detected in J4B anthers (Figure 4C), while high expression was detected in the tapetum of J4A anthers (Figure 4D).

### 2.4. The Effect of orf116b Expression in E. coli and Cotton

**Figure 5:**
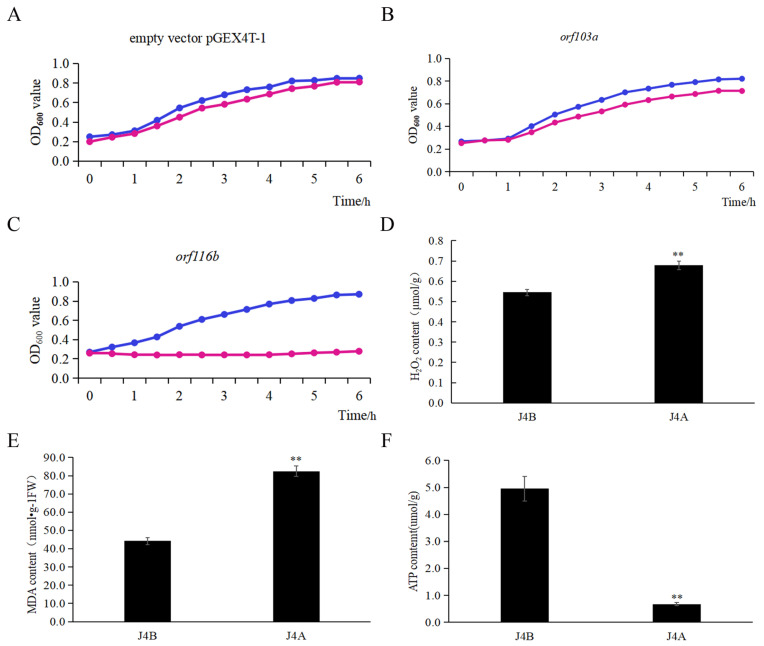
Effect of *orf103a* and *orf116b* expression in *Escherichia coli* cells and biochemical characterization of J4A and J4B anthers. Growth of *E. coli* cells carrying (**A**) the pGEX4T-1 empty vector and the (**B**) pGEX4T-1-*orf103a* and (**C**) pGEX4T-1-*orf116b* constructs without and with IPTG induction. Blue lines represent the growth curve of *E. coli* under IPTG induction; red lines represent the growth curve of *E. coli* without IPTG induction. The growth of the transformed *E. coli* cells was assessed by measuring OD_600_ every hour for six hours. (**D**) H_2_O_2_ content, (**E**) MDA content, and (**F**) ATP content in the J4A and J4B anthers. The error bars represent the standard deviation of three replicates, and ** indicates a significant difference compared to the control (J4B) at *p* < 0.01 (Student’s *t*-test).

Without isopropyl β-D-1-thiogalactopyranoside (IPTG) induction, the growth pattern of *E. coli* BL21 (*DE3*) cells carrying the candidate CMS gene *orf116b* was similar to that of the two controls, the *E. coli* cells carrying *orf103a* (mt transcript control) and the *E. coli* cells containing the pGEX4T-1 empty vector. However, after adding IPTG and incubating for 6 h at 18 °C, the *E. coli* cells carrying *orf116b* showed obvious growth retardation compared to these two controls (Figure 5A–C), indicating a toxic effect of *orf116b* expression on bacterial growth.

Furthermore, to investigate the role of *orf116b* in regulating anther vigor in J4A and J4B, we analyzed the reactive oxygen species (ROS) [hydrogen peroxide (H_2_O_2_) and malondialdehyde (MDA) ] and ATP levels in the mature anthers of the two lines. J4A had higher H_2_O_2_ and MDA levels but a lower ATP content than J4B (Figure 5D–F), indicating that the disruption of mt ROS homeostasis and an insufficient supply of ATP led to male sterility in J4A.

### 2.5. Orf116b Probably Interacts with an Anther-Specific Protein LAT52 In Vitro

**Figure 6:**
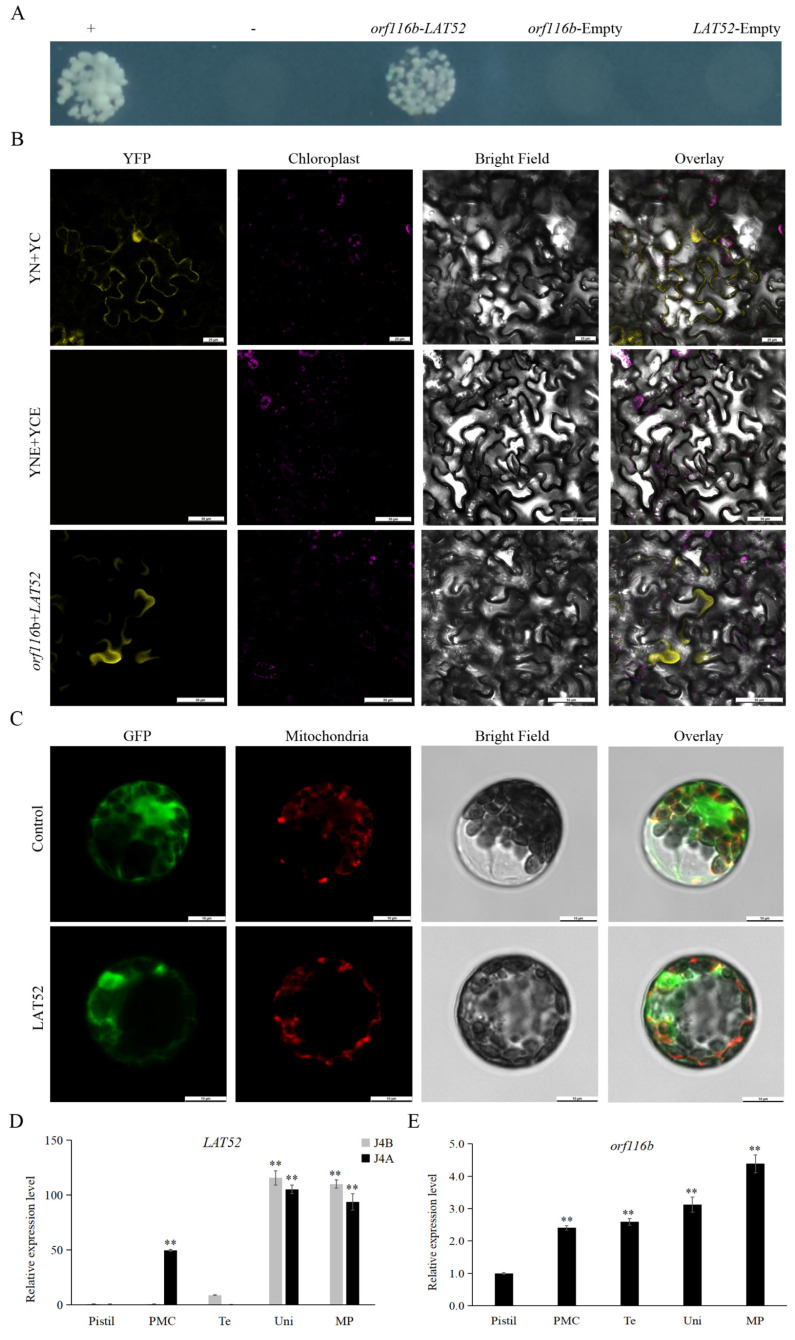
Analysis of orf116b-LAT52 interaction and *LAT52* and *orf116b* gene expression patterns. (**A**) Split-ubiquitin yeast two-hybrid assay shows the interaction of orf116b with LAT52 in yeasts. +, Positive control; −, negative control. (**B**) BiFC assay shows orf116b interaction with LAT52 in tobacco leaves. YNE + YCE, empty vector negative control; YN + YC, positive control; YFP, yellow fluorescent protein. Scale bars of (**B**) correspond to 50 μm. (**C**) Subcellular localization of LAT52 in Arabidopsis protoplasts. Green represents the green fluorescent protein (GFP) signal; bright red indicates the mitochondrial localization signal; orange-yellow and orange indicate the overlap of the green fluorescent signal and the red fluorescent signal; in other words, orange-yellow and orange indicate mitochondrial localization. Scale bars of (**C**) correspond to 10 μm. (**D**) The relative expression levels of the *LAT52* in J4A and J4B pistils and anthers at different developmental stages. The error bars represent the standard deviation of three replicates, and ** indicates a significant difference compared to the control (relative expression levels in J4B’ pistil) at *p* < 0.01 (Student’s *t-*test). (**E**) The relative expression levels of *orf116b* in J4A anthers at different developmental stages within J4A. The error bars represent the standard deviation of three replicates, and ** indicates a significant difference compared to the control (relative expression levels in the pistil) at *p* < 0.01 (Student’s *t-*test). PMC, pollen mother cell stage; Te, tetrad period; Uni, uninucleate stage; MP, mature pollen grain stage.

Initial analysis showed that the CMS protein orf116b has a transmembrane structure, with its N-terminal and C-terminal located within the membrane (Figure S3A). Therefore, we used a split-ubiquitin membrane-based yeast two-hybrid (MYTH) system to screen the proteins that probably interact with orf116b. After the preliminary screening, we identified 96 candidate proteins that could interact with orf116b and obtained the putative monoclonal yeast colonies carrying these candidates (Figure S4). From there, we performed PCR amplification with the Yeast Colony Rapid Detection Kit (Nanjing Ruiyuan Biotechnology Co., Ltd., Nanjing, China, RY8001) on all of them. Following sequence alignment with Seqman (https://www.dnastar.com/software/lasergene/seqman-ultra/, accessed on 16 September 2023) and BLAST (https://www.novopro.cn/blast/blastn.html) comparison, we ultimately obtained 49 distinct sequences. As a result, 22 out of these 49 grew on SD-Trp/Leu/His (SD-TLH) and SD-Trp/Leu/His/Ade (SD-TLHA) selection media (Figure S5, Table S6), suggesting interactions with orf116b. From these 22 candidates, genes encoding the ribosome biology protein NOP53 (LOC107931849), the anther-specific protein LAT52-like (LOC107942900), and the NAC domain-containing protein seventeen translation variant X4 (LOC107914072) were selected for further analysis. In the MYTH assay, colonies carrying the anther-specific protein LAT52 alone grew on SD-TLHA medium, suggesting most likely interaction with orf116b (Figure 6A).

Furthermore, in the BiFC assay performed to assess the interaction of LAT52 with orf116b, the tobacco cells co-transfected with orf116b and LAT52 displayed yellow fluorescence under a laser confocal microscope (Figure 6B), suggesting an interaction between orf116b and LAT52. Subsequently, subcellular localization analysis was performed by transfecting the protoplasts obtained from Arabidopsis seedlings with the LAT52-GFP-pAN580 plasmid. Here, the LAT52-GFP-specific green fluorescence was detected in the Arabidopsis protoplast, including mt (Figure 6C), indicating that LAT52 is expressed in the mt.

Subsequent, quantitative real-time polymerase chain reaction (qRT-PCR)-based analysis showed a lack of *LAT52* expression in J4B anthers during the pollen mother cell stage, but a significantly high *LAT52* expression in J4A (Figure 6D). In addition, *orf116b* expression was detected in J4A anthers from the pollen mother cell stage; the expression gradually increased with the development of anthers (Figure 6E). These observations revealed an overlap in the expression of *LAT52* and *orf116b* in the anthers of J4A during the pollen mother cell stage (Figure 6D,E), suggesting a probable interaction between orf116b and LAT52.

### 2.6. Overexpression of orf116b in Rice, Arabidopsis, Tobacco, and Cotton Led to Pollen Sterility

**Figure 7:**
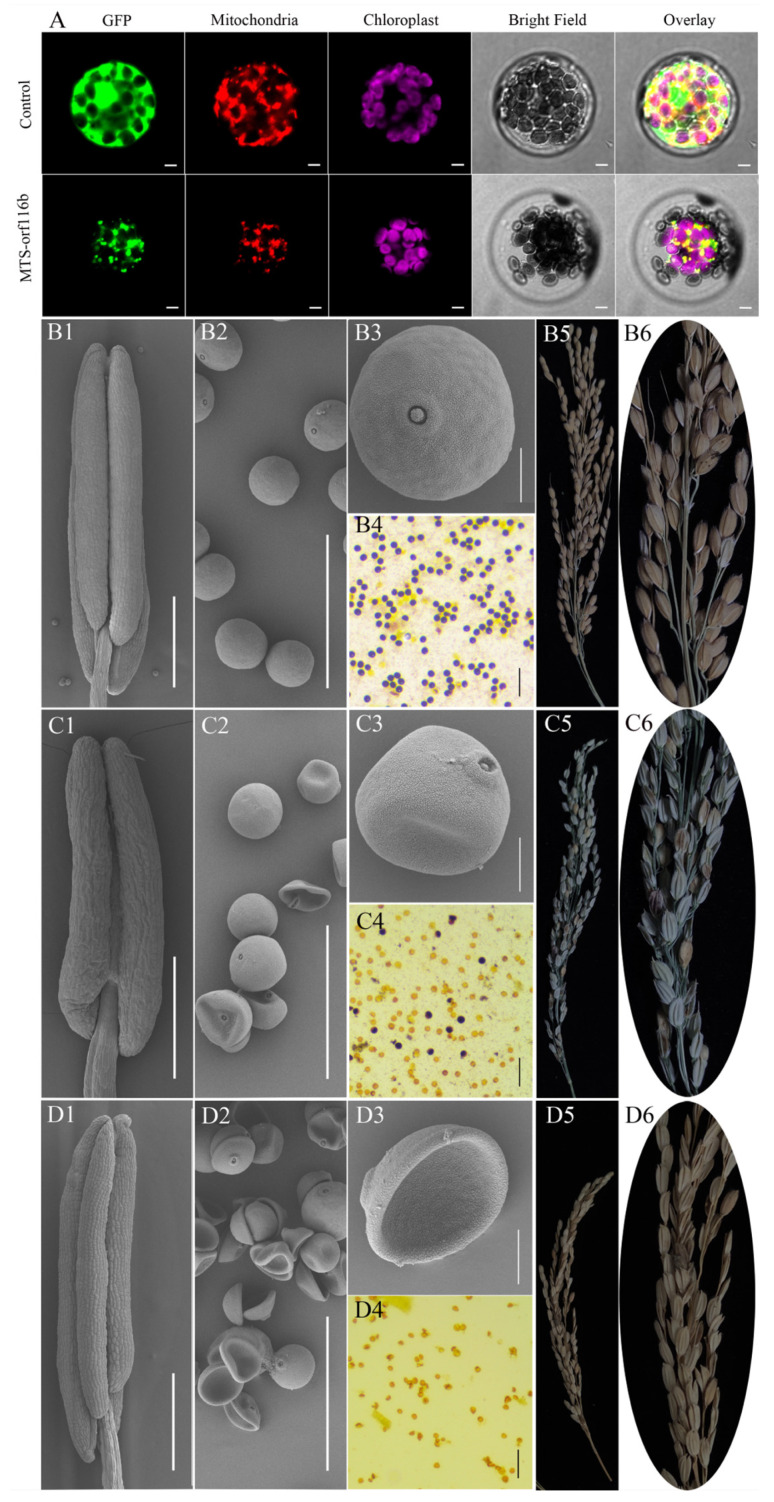
Ectopic overexpression of *orf116b* leads to partial and complete sterility in rice. (**A**) Subcellular localization of MTS-*orf116b* in Arabidopsis protoplasts demonstrates its expression within the mitochondria. Green represents the green fluorescent protein (GFP) signal; bright red indicates the mitochondrial localization signal; magenta indicates the autofluorescence of chloroplasts; orange-yellow and orange indicate the overlap of the green fluorescent signal and the red fluorescent signal. Scanning electron microscopic images of anthers in the wild-type rice. Scale bars of (**A**) correspond to 20 μm. (**B1**–**B3**), the partial sterile OE-MTS-*orf116b* rice (**C1**–**C3**), and the completely sterile OE-MTS-*orf116b* rice (**D1**–**D3**). I_2_-KI staining of pollen grains of the wild-type rice (**B4**), the partially sterile OE-MTS-*orf116b* rice (**C4**), and the completely sterile OE-MTS-*orf116b* rice (**D4**). Panicles of the wild-type rice (**B5**), the partial sterile OE-MTS-*orf116b* rice (**C5**), and the completely sterile OE-MTS-*orf116b* rice (**D5**). Seeds of the wild-type rice (**B6**), the partial sterile OE-MTS-*orf116b* rice (**C6**), and the completely sterile OE-MTS-*orf116b* rice (**D6**). Scale bars of (**B1**,**C1**,**D1**) correspond to 500 μm; scale bars of (**B2**,**C2**,**D2**) correspond to 100 μm; scale bars of (**B3**,**C3**,**D3**) correspond to 20 μm; scale bars of (**B4**,**C4**,**D4**) correspond to 200 μm.

To verify the role of *orf116b* in regulating pollen sterility in the CMS line, we constructed the MTS-*orf116b*-GFP-pAN580 plasmid (*orf116b* fused with the mitochondrial targeting signal peptide, MTS) in vectors driven by the ubiquitin promoter and CaMV::35S promoter for genetic transformation of monocot (rice) and dicot (Arabidopsis, tobacco, and cotton) species, separately. Subcellular localization analysis in Arabidopsis protoplasts revealed MTS-*orf116b* expression in the mt (Figure 7A). Subsequently, we obtained the rice, Arabidopsis, tobacco, and cotton transgenic plants overexpressing MTS-*orf116b* (OE-MTS-*orf116b*) to assess the specific role of *orf116b* in inducing male sterility in these higher plants.

In rice, the wild-type plants had smooth and plump anthers (Figure 7B1) and pollen grains (Figure 7B2,B3); these plants displayed blue-purple mature pollen grains after I_2_-KI staining (Figure 7B4) and had plump and abundant seeds (Figure 7B5,B6). On the contrary, out of the 15 OE-MTS-*orf116b* transgenic rice plants generated, four had a few active pollens (partially sterile) (Figure 7C1–C6), five were completely sterile (Figure 7D1–D6), and the remaining six exhibited normal fertility (Figure 7B1–B6). Particularly, the male sterile rice plants (Figure 7C1–C6,D1–D6) exhibited deformed or shrunk anthers (Figure 7C1,D1) with hollow (Figure 7C2,C3) and depressed (Figure 7D2,D3) pollen grains, without any blue-purple color after I_2_-KI staining (Figure 7C4,D4). The partially sterile transgenic rice plants produced seeds with empty shells and a few normal seeds (Figure 7C5,C6), while the completely sterile ones produced only empty shells with no functional embryos (Figure 7D5,D6). These results indicated a significant role for orf116b in regulating anther abortion in rice.

**Figure 8:**
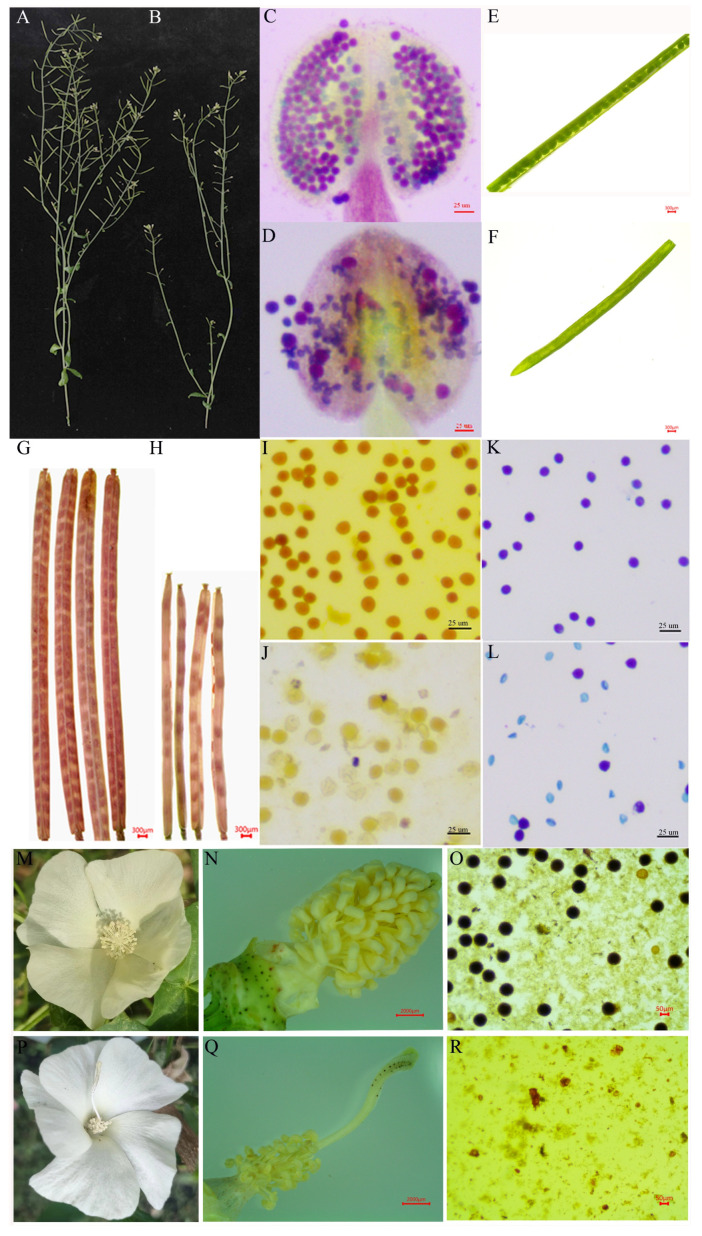
Overexpression of *orf116b* causes partial and complete sterility in Arabidopsis, tobacco, and cotton. The main branch of the wild-type (**A**) and OE-MTS-*orf116b* transgenic (**B**) Arabidopsis plants. Alexander staining of the pollen of the wild-type (**C**) and OE-MTS-*orf116b* transgenic Arabidopsis plants (**D**) at the full bloom stage. Pods of the wild-type (**E**) and OE-MTS-*orf116b* transgenic Arabidopsis plants (**F**) at the pod formation stage. Pods of the wild-type (**G**) and OE-MTS-*orf116b* transgenic (**H**) Arabidopsis plants at seed maturity. I_2_-KI staining of the pollen of the wild-type (**I**) and CaMV::35S-*orf116b*-GFP transgenic (**J**) tobacco plants. Alexander staining of the pollen of the wild-type (**K**) and CaMV::35S-*orf116b*-GFP transgenic (**L**) tobacco plants. Flowers of the wild-type (**M**) and OE-MTS-*orf116b* transgenic (**P**) cotton plants at pollen maturity. Anthers and stigmas of the wild-type (**N**) and OE-MTS-*orf116b* transgenic (**Q**) cotton plants at pollen maturity. I_2_-KI staining of the pollen of the wild-type (**O**) and CaMV::35S-*orf116b*-GFP transgenic (**R**) cotton plants.

In Arabidopsis, compared to the wild-type plants with normal fertility (Figure 8A,C,E,G), the plants overexpressing *orf116b* (ectopic; OE-MTS-*orf116b*) displayed semi-sterility (Figure 8B,D,F,H). Phenotype analysis (Figure 8A,B) and Alexander staining (Figure 8C,D) revealed that the pollens of 45 OE-MTS-*orf116b* transgenic plants were partially sterile. The pods formed by these transgenics (Figure 8F,H) were significantly smaller than those of the wild-type plants (Figure 8E,G) at seed maturity. Furthermore, the transgenic plants (Figure 8H) had fewer seeds than the wild-type plants (Figure 8G).

In tobacco, the pollen grains of the wild-type plants were fertile and appeared dark brown after I_2_-KI staining and blue-purple after Alexander staining (Figure 8I,K), indicating pollen vitality. Meanwhile, the pollen grains of the OE-MTS-*orf116b* transgenic plants were deformed and appeared shrunk, with a light brown color after I_2_-KI staining (Figure 8J). Only a few OE-MTS-*orf116b* transgenic tobacco pollen grains were faintly stained after I_2_-KI and Alexander staining. Moreover, the pollen grains were smaller and irregular, indicating sterility (Figure 8L).

In cotton, compared to the wild-type plants with normal stigma and fertile pollen grains (Figure 8M–O), the 12 transgenic plants overexpressing *orf116b* (OE-*orf116b*) showed abnormally longer stigma (about two times longer than the wild type) (Figure 8P,Q) and a very few deformed pollen grain remnants (Figure 8R). These observations in the transgenic plants prove the critical role of orf116b in determining CMS.

## 3. Discussion

### 3.1. Excessive Proliferation of Tapetal Cells Leads to the Inability to Form Microspores

The tapetum, a specialized somatic cell layer within the anther wall, undergoes PCD and degradation at a specific stage and plays a pivotal role in the development of microspores. This process is essential for providing the microspores with the requisite energy, proteins, lipids, and enzymes for their development. However, any deviation from the normal situation, such as excessive proliferation or premature or delayed degradation of tapetal cells, can interrupt microspores’ development and lead to male sterility [26,27]. In maize, Moon et al. found that the CMS gene *ms32* is specifically expressed in the tapetum during the meiotic phase. Moreover, the *ms32-ref*-*mac1-1* double mutant could not form the tapetal precursors and exhibited excessive somatic cell proliferation, forming disordered cell layers [28]. Consistent with this fact, the present study found that in the sterile line J4A, the tapetal cell layer experiences excessive proliferation during meiosis (Figure 1R,S), preventing PCD. This change causes a deficiency of energy and nutrients required for microspore development; thus, even after meiosis, the microspores fail to form the tetrad microspores (Figure 1H1,T), ultimately resulting in sterility due to the absence of pollen grains. Thus, our findings confirm that excessive proliferation of tapetal cells causes CMS by hindering microspore formation.

### 3.2. Orf116b’s Toxicity Disrupts the mt Function, Leading to a Decrease in ROS and ATP Syntheses

In plants, rearrangement of the mt genome generates CMS genes, which co-transcribe with the functional genes as new chimeric ORFs and perform novel functions [29,30]. The present study found that the CMS line J4A and its cytoplasmic nuclear homologous maintainer line J4B shared a high identity. High-quality sequencing of their mt genomes identified a novel CMS gene *orf116b* in J4A. We found that orf116b co-transcribed with the *rpl2* and *rpl5* genes, encoding ribosomal large subunit proteins in the mt in the CMS line J4A, adhering to a CMS gene’s characteristics. Several studies have reported the co-transcription of CMS genes with functional genes, such as the co-transcription of *orf610a* with *atp1* in cotton CMS-D2 ZBA [11] and *orf346* with *nad3* and *rps12* in rape Nsa CMS [17]. Thus, consistent with these earlier reports, the present study’s observations suggest similarities in CMS regulatory mechanisms across plant species. Moreover, the genes *rpl2* and *rpl5* found to co-transcribe with the CMS gene in this study are known to influence the structure of the mt inner membrane through translational mechanisms. A recent study reported that an abnormal sequence of the mt ribosome genes *rps4* and *rpl10* damaged the mt membrane, resulting in abortion in the cotton line LD6A [31]. Consistent with this, we also detected decreased ribosomal protein (RP) content (Figure S6) and deformed and ruptured tapetal mt in J4A (Figure 1H2). Thus, the present study establishes a strong link between the genes *orf116b*, *rpl2*, and *rpl5* and the mt structure.

CMS genes are known to impair mt function by expressing toxic proteins, which disrupt the normal function of enzymes associated with respiratory chain; this effect subsequently leads to male sterility [32,33]. Various chimeric CMS genes, such as cotton *orf610a* [11], rice *orf352* [5], maize *orf355* [14], wheat *orf256* [18], and oilseed rape *orf346* [17], have demonstrated toxicity in prokaryotic systems [25]. Reactive oxygen species (ROS) can act as signaling molecules, and maintaining their basal levels is essential for plant development [24,34]. The mt dysfunction, in turn, leads to excessive ROS production, causing CMS [35,36]. Similarly, the present study demonstrated that orf116b suppressed the growth of *E. coli* (Figure 5C). Concurrently, a reduction in ATP levels (Figure 5F) and an excessive accumulation of ROS (Figure 5D,E) were observed within the anthers of the sterile line J4A. Therefore, we speculate that orf116b possesses toxicity and disrupts mt function, leading to decreased ATP synthesis and the overproduction of ROS. We also assume that ROS in J4A may acts as one of the mt signals that enter the nucleus and regulate gene expression (retrograde signaling) during the meiosis of microspores, which in turn affects the mt via anterograde signaling (nucleus to mitochondria). However, this complex mechanism needs to be investigated in detail.

### 3.3. LAT52 Probably Affects Pollen Development in J4A

The MYTH system mediated by split-ubiquitin is generally used to detect the interaction between proteins in the cytoplasm or on membranes [37,38,39]. Since the genes responsible for CMS encode transmembrane proteins [5,11,18], we used the MYTH system to screen the interacting membrane proteins. This approach helped us identify that LAT52 interacts with orf116b, which was confirmed via BiFC (Figure 6B). Meanwhile, bioinformatics (Figure S7) and subcellular localization (Figure 6C) analyses indicated the localization of LAT52 in mitochondria. These results collectively suggested that orf116b could specifically interact with LAT52 in cotton.

**Figure 9:**
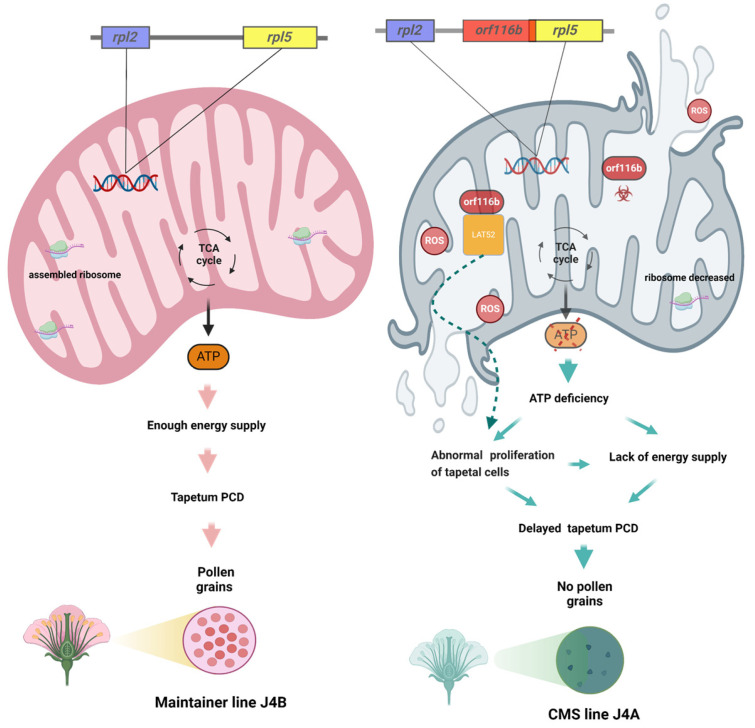
The schematic diagram shows the *orf116b* module-based mechanism inducing pollen abortion in the cotton line J4A. As per the proposed model, the rearrangement of the J4A mt genome produces a new gene, *orf116b*. Orf116b protein is toxic and adversely affects mt function, leading to mt dysfunction with excessive ROS accumulation and reduced ATP production. *Orf116b* triggers abnormal proliferation and delayed PCD of tapetal cells in anthers, leading to pollen sterility. Orf116b also appears to interact with an anther-specific protein, LAT52, in the mt and contributes to CMS in J4A; however, this mechanism needs further investigation.

Research has identified LAT52 as a small extracellular Cys-rich and heat-stable glycosylated, anther-specific protein participating in pollen development and pollen tube growth by regulating pollen hydration and/or germination [40,41,42]. Multiple upstream cis-regulatory elements regulate the *LAT52* gene by precisely acting at the transcriptional level. Thus, the absence of the anther-specific *LAT52* can lead to pollen infertility, highlighting its indispensable role in the reproductive process [43]. In tomatoes, LAT52 transcripts are found after microspore mitosis; this gene’s expression gradually increases with microspore development and peaks at pollen maturity [44]. LAT52 transcripts and protein also assist in pollen tube growth at the mature pollen stage. However, little is known about LAT52 in other plants. In this study, gene expression analysis indicated an overlap in *LAT52* and *orf116b* expression in the anthers of J4A during the pollen mother cell stage (Figure 6D,E), and the subcellular localization showed that portions of LAT52 (Figure 6C) and orf116b (Figure S8) are located in the mt. Additionally, the semi-thin sections of anthers showed excessive proliferation of tapetal cells during the late pollen mother cell stage (Figure 1R). Earlier, Dewey et al. found that the CMS protein ORFH79 interacts with P61, causing dysfunction in mt energy production and abnormality in pollen development in the CMS rice line Yuetai A [16]. Therefore, we speculate that LAT52 probably contributes to CMS in J4A by interacting with orf116b during the late pollen mother cell stage and inducing abnormal proliferation of the tapetal cells (Figure 9). These results reveal the potential of the orf116b module in hybrid seed production in cotton and other crops. However, more experiments, including functional validation of LAT52, are needed to confirm the CMS gene-mediated mechanisms in cotton.

### 3.4. Proposed Mechanism of Pollen Abortion in Cotton

The present study found *orf116b* expression as early as the pollen mother cell stage (in situ hybridization; Figure 4D), and the start of abnormal proliferation in the tapetal cells initiated abnormal proliferation at the late pollen mother cell stage (Figure 1R) in the sterile line J4A. The J4A tapetal cells exhibited proliferation subsequently during the meiotic phase and transformed into small, disordered multilayered cells (Figure 1S), reducing PCD. Based on all these observations, we speculated that the toxicity of orf116b (Figure 5A–C) and the co-transcription of *orf116b* with *rpl2* and *rpl5* (Figure 4A,B) led to mt membrane damage (Figure 1H1), affecting microsporogenesis and resulting in early abortion. The excessive accumulation of ROS (H_2_O_2_ and MDA) found accompanying the mt membrane damage (Figure 5D,E) also led to early degradation of the tapetal layer [5,11] or delayed PCD in the tapetal layer [24,45]. Furthermore, research has established that an equilibrium in ROS is critical for pollen development [33,46]. The mt-derived ROS serve as intrinsic signals for plant cell proliferation and differentiation [28] and a feedback signal to regulate gene expression [47]. Recently, Zheng et al. found that, in the distant polyploid male sterile line of cotton (LD6A), the downregulation of histone acetyltransferase (*HATs*) decreased ROS scavenging and accumulation, damaging the mt membrane and impairing micropore development [31]. Thus, our findings suggest that *orf116b* triggers abnormal proliferation of tapetal cells, delays PCD, damages the mt membrane, and alters ROS homeostasis and energy supply (ATP deficiency), resulting in a pollen-free type of male sterility in J4A (Figure 9).

## 4. Materials and Methods

### 4.1. Plant Materials and Growth Conditions

A cotton (*Gossypium hirsutum* L.) CMS line, J4A [48], and its maintainer line, J4B, were used in this study. The two lines were grown in the Guangxi University experimental farm (22.8° N, 108.2° E; Guangxi, China) from April to October of 2021 and the National Wild Cotton Nurseryin Nanpi Farm (38.0° N, 116.7° E; Hainan, China) from October to March of 2021.

### 4.2. Analysis of J4B and J4A Anther Phenotypes

Flowers at the full blooming stage were collected from the cotton plants to analyze pollen viability and morphology. Flower buds of different sizes were collected, and the diameter of each bud was measured using a Vernier caliper. Each flower was then carefully crushed to extract the anthers, and the pollen grains were collected and stained with a 1% I_2_-KI solution to assess the viability. In addition, flower buds collected at the pollen mother cell stage (2–3 mm diameter), tetrad stage (3–4 mm diameter), and mature pollen formation stage (>5 mm diameter) were analyzed by scanning electron microscopy (SEM) (Hitachi, Ltd., Tokyo, Japan, Model SU8100) and transmission electron microscopy (TEM) (Hitachi, Ltd., Tokyo, Japan, Model HT7800) [49]. All flower buds were collected on the sampling dates at 10:00 in the morning.

Anther degeneration was meticulously examined at the cytological level using an enhanced semi-thin sectioning approach [50]. First, the anthers collected at the pollen mother cell stage, late pollen mother cell stage, meiosis stage, and tetrad stage were fixed in 1% osmic acid (Ted Pella Inc., Redding, CA, USA, 18456, Ted Pella, Inc.) at room temperature for 7 h. These anthers were further rinsed in 0.1 M phosphate buffer (Servicebio, G0002, https://www.servicebio.cn/goodsdetail?id=20378, accessed on 9 December 2023) (pH 7.4) and dehydrated in 30%, 50%, 70%, 80%, 95%, and 100% ethanol sequentially for 1 h each and 3:1, 1:1, and 1:3 anhydrous ethanol: acetone mixtures for 0.5 h each. Then, the anthers were permeated in 3:1, 1:1, and 1:3 mixtures of acetone and the 812 embedding agent (SPI, 90529-77-4, https://us.vwr.com/store/product/12359781/embed-812-embedding-kit-electron-microscopy-sciences, accessed on 12 January 2024 [16]) by incubating at 37 °C for 4 h, 12 h, and 4 h, respectively, and in pure 812 embedding agent overnight in a 37 °C oven. Further, the anther tissues were polymerized by incubating in an oven at 60 °C for 48 h and sectioned to 1.5 µm thickness using an automated microtome (Leica Biosystems, Leica HistoCore Nanocut R, https://www.leicabiosystems.com/zh/research/research-microtomes/histocore-nanocut-r/, accessed on 16 January 2024). These sections were stained with toluidine blue solution (Servicebio, G1032, https://www.servicebio.cn/, accessed on 16 January 2024), incubated at 60 °C for 2 min, washed with water, differentiated with 95% alcohol, dried at room temperature, and sealed with neutral gum. Finally, the stained sections were observed under a microscope (NIKON ECLIPSE CI, Nikon, Japan, https://www.microscope.healthcare.nikon.com/zh_CN/products/upright-microscopes/eclipse-ci-series, accessed on 17 January 2024).

### 4.3. DNA Extraction, Sequencing, and Genome Assembly

The seeds of J4A and J4B cotton lines collected from the Yazhou farm of the Cotton Research Institute (the Chinese Academy of Sciences, Hainan, China) were delinted using concentrated sulfuric acid, placed onto wet gauze in a culture dish, and kept in an incubator without light at 25–28 °C for a day. The germinated seeds were transferred to a tap-water hydroponic system and kept in a dark incubator at 25–28 °C for seven days to obtain etiolated seedlings.

Total DNA was extracted from 0.5 g of the etiolated seedlings using the modified cetyltrimethylammonium bromide (CTAB) method [51], and the purity and quantity of the extracted DNA were assessed with Nanodrop 2000 (Thermo Fisher Scientific, Carlsbad, CA, USA) and Qubit 2.0 (Thermo Fisher Scientific, Carlsbad, CA, USA). Then, the DNA was sequenced (accession number: PRJNA934044) following the second-generation Illumina Hi-seq and third-generation PacBio methods.

Approximately 1 µg of high-quality total genomic DNA extracted from the etiolated seedlings was used to construct a DNA library with the TruSeq™ Nano DNA Sample Prep Kit (Illumina), following the manufacturer’s instructions. The library was sequenced on an Illumina NovaSeq 6000 system (BIOZERON Co., Ltd., Shanghai, China) to generate paired-end reads (150 bp). Subsequently, high-quality data were obtained from the raw data by trimming reads with adapter sequences, reads containing non-AGCT at the 5′ end, reads of low quality (<Q20), reads containing 10% unknown (N) and repetitive sequences, and short fragments less than 75 bp after adapter and quality processing using Trimmatic v0.3 (http://www.usadellab.org/cms/index.php?page=trimmomatic, accessed on 18 November 2022).

Another 5 µg of the high-quality total genomic DNA was sheared and concentrated using BluePippin cassettes (Sage Science, Beverly, MA, USA), ligated to approximately 20 kb SMRTbell library (PacBio, Menlo Park, CA, USA), and sequenced on a PacBio Sequel II system (PacBio, Menlo Park, CA, USA). The raw data obtained from third-generation sequencing platform were filtered using Trimmomatic v0.39 (http://www.usadellab.org/cms/index.php?page=trimmomatic, accessed on 20 December 2022), which removes reads shorter than 200 bp, reads with adapter sequences, and subreads shorter than 200 bp. Then, the Illumina reads were assembled using GetOrganelle v1.7.1 (https://github.com/Kinggerm/GetOrganelle, accessed on 27 December 2022) and compared with the PacBio data using BWA v0.7.17 (https://github.com/lh3/bwa, accessed on 29 December 2022). The third-generation sequencing data were extracted, mixed with the Illumina data, and assembled using SPAdes v3.14.1. Further, the sequences with sufficient coverage and assembly length were selected as candidates, and the mt scaffold sequences were confirmed by comparing them with the Nuclear DNA library. The clean reads were aligned to the mt genome sequence, and the bases were corrected using Pilon v1.23. Finally, the start codon and the coding direction of the mt scaffolds were determined based on the reference genome (GenBank Acc. No. NC_027406.1), and the mt genome sequence was obtained. The circular map of the mt genome was constructed based on the assembled sequence, and the coding genes were predicted using OGDRAW (https://chlorobox.mpimp-golm.mpg.de/OGDraw.html, accessed on 11 January 2023).

### 4.4. Annotation of the mt Genome

The GeSeq online tool [52] was used with default parameters to predict the protein-coding genes, transfer RNA (tRNA), and ribosome RNA (rRNA) in the mt genome sequence. The position of each coding gene was determined following a BLAST search against the reference mt genes of upland cotton (GenBank Acc. No. NC_027406.1). Further, the start/stop codons and the intron/exon boundaries were manually corrected using SnapGene Viewer v5.0.5, and highly accurate conserved gene sets were obtained. Then, the sequences of the mt protein-coding genes, including the conserved mt genes and the ORFs, were compared with the public databases using BLASTP (<1 × 10^−5^). Finally, the amino acid sequences of the J4A and J4B mt-coding genes were compared with the NCBI non-redundant (Nr) protein database, Swiss-Prot, Clusters of Orthologous Groups (COGs, http://www.ncbi.nlm.nih.gov/COG/, accessed on 13 January 2023), Kyoto Encyclopedia of Genes and Genomes (KEGG, http://www.genome.jp/kegg/, accessed on 13 January 2023), and Gene Ontology (GO, http://www.geneontology.org/, accessed on 13 January 2023) for functional annotation.

### 4.5. Screening and Validation of CMS Candidate Genes

Due to the potential association of new ORFs with CMS [11,25,53], we analyzed the positioning and transmembrane domains of all ORFs in the J4A mt genome, and then categorized these ORFs into three types: (1) ORF unique to the mt genome of J4A, which are absent from the mt genome of J4B; (2) ORF present close to an mt protein-coding gene (within a region 500 bp upstream or downstream); and (3) ORF with local transmembrane domain (TMHMM Server v2.0). After identification, RT-PCR was performed to verify the co-transcription of the novel CMS gene (*orf116b*) and their nearby mt protein-coding genes (*rpl2* and *rpl5*). The reaction was carried out using the cDNA obtained from the anthers of J4B and J4A at the mature pollen grain stage, I-5 2xHigh Identity Master Mix (TP001; Tsingke Biotechnology Co., Ltd., Beijing, China), and primers designed across the ORFs and its upstream and downstream genes. The primers 2O F and 2O R were used to verify *orf116b*-*rpl2* co-transcription, and 5O F and 5O R were used to verify *orf116b*-*rpl5* co-transcription (Table S7).

### 4.6. qRT-PCR-Based Analysis

The pistils and anthers (four types: mother cell stage (PMC), tetrad stage (Te), uninuclear stage (Uni), and mature pollen grain stage (MP)) were collected from the J4A and J4B cotton lines at full blooming stage. These samples were frozen in liquid nitrogen immediately after collection and stored at −80 °C. Total RNA was extracted from the sample with the Universal Polysaccharide and Polyphenol Universal Plant RNA Extraction Kit (Huayueyang Biotechnology Co., Ltd., Beijing, China) and reverse-transcribed into cDNA using the M-MLV Reverse Transcriptase Kit (ZR102, Zoman Biotechnology Co., Ltd., Beijing, China). Then, qRT-PCR was performed using the cDNA in a 15 µL reaction mixture with ChamQ SYBR qRT-PCR Master Mix (Nanjing Vazyme Biotech Co., Ltd., Nanjing, China) on a Roche Light Cycler 480 II to analyze the expression of the CMS candidate genes. The relative expression levels of the candidate genes were determined using the upland cotton *18S* rRNA gene as the internal reference and following the 2^−ΔΔCt^ method [54]. The primers used in this assay are shown in Table S1.

### 4.7. Expression of orf116b in a Prokaryotic System

Two CMS-related genes, *orf116b* and *orf103a* (Figure 3E), were selected, amplified with primers designed using CE Design v1.04 (Table S7), and ligated into a prokaryotic expression vector pGEX4T-1 using the ClonExpress II One Step Cloning Kit (Vazyme Biotech Co., Ltd., Nanjing, China). *Escherichia coli* BL21 (*DE3*) competent cells (Zoman Biotechnology Co., Ltd., Beijing, China) were transformed using these constructs following the manufacturer’s instructions, and the transformed cells were cultured in Luria Bertani (LB) liquid medium with ampicillin. About 1 mL of the culture of *E. coli* (pGEX4T-1-*orf116b*, pGEX4T-1-*orf103a*, or the pGEX4T-1 empty vector) was inoculated (1%) into 100 mL of LB broth (ampicillin) and incubated at 37 °C in a shaker at 200 rpm. At an OD600 of 0.6, isopropyl β-D-1-thiogalactopyranoside (IPTG; 1 mM) was added to induce protein expression, maintaining a non-induced culture as a control. Every 30 min, 3 mL of the bacterial culture was collected in a cuvette, and the absorbance at 600 nm was measured; three replicate samples were analyzed at each time point. Finally, the growth curve was plotted using the OD_600_ values recorded for a total of six hours.

### 4.8. In Situ Hybridization Analysis of orf116b in Cotton

The anthers of J4B and J4A cotton lines at the pollen mother cell stage were fixed in a plant tissue fixative for more than 12 h, dehydrated in gradient alcohol, cleared in absolute alcohol and xylene, embedded in wax, and cut into sections of 6 µm thickness using a slicer (Leica, RM2016). After digestion with protease K (20 µg/mL), these tissue sections on the slides were hybridized to a probe (TACAGTAGAGGAGAGTACGAAATCAGT), blocked with rabbit serum, and incubated with anti-DIG-AP antibody at 40 °C for 50 min. The sections were then washed with Tris-buffered saline (TBS; four times, 5 min each), incubated in BCIP/NBT solution, and sealed with glycerol gelatin for microscopic analysis (NIKON ECLIPSE CI, Nikon, Japan) and image acquisition. This assay was carried out as per the procedure in the instruction manual of the AP-labeled mouse anti-digoxin antibody (anti-DIG-AP) Kit (Jackson, 200-052-156).

### 4.9. Determination of Anther Physiological and Chemical Indexes

Anthers at the mother cell stage, tetrad stage, uninuclear stage, and mature pollen grain stage were collected from J4A and J4B cotton lines at the full blooming stage, peeled on ice, and used to analyze various indexes. The hydrogen peroxide (H_2_O_2_) and malondialdehyde (MDA) contents were measured using the plant H_2_O_2_ enzyme-linked immunosorbent assay (ELISA) kit and the MDA ELISA kit, respectively. Then, the adenosine triphosphate (ATP) content was determined using an ATP kit, and the ribosomal protein (RP) content was determined using an RP kit. All kits were purchased from the Enzyme-Linked Biotechnology Co., Ltd. (Shanghai, China), and the index values were measured following the manufacturer’s instructions. The data collected were subsequently analyzed using IBM SPSS Statistics version 28.0.1.0 (IBM Corp., 2021) to determine the statistical significance of differences between the lines, with the significance level set at *p* < 0.01 (Student’s *t*-test) [55].

### 4.10. Subcellular Localization Analysis of orf116b and LAT52

Subcellular localization was carried out by transforming Arabidopsis protoplasts with the MTS-*orf116b*-GFP (carrying CaMV::35S-mt targeting sequence), *orf116b*, and *LAT52*-GFP constructs [56]. Here, the GFP-pAN580 empty vector served as the control. First, these constructs and the control vector were co-transformed with the mt localization vector (Mkate carrying the mt localization signal protein (mtsp)) [57] into the protoplasts and incubated in the dark at 28 °C for 18–24 h. Then, the protoplasts were observed under a laser confocal microscope (Nikon C2-ER, Tokyo, Japan) at 488/510 nm excitation/emission wavelengths to detect the GFP protein, 561 nm/580 nm to detect the mt-localized fluorescent protein, and 640 nm/675 nm to detect the chloroplast’s spontaneous fluorescence.

### 4.11. Overexpression of orf116b in Rice, Arabidopsis, Tobacco, and Cotton

For transforming rice, the mt targeting sequence *Rf1b* 5′ (MTS) [5] was added upstream of the start codon of *orf116b* and ligated into the pRHVcGFP vector. The obtained UBI1::MTS-*orf116b*-GFP construct was further inserted into *Agrobacterium tumefaciens*. These Agrobacterium cells were used to transform the embryogenic callus obtained from the japonica cultivar Zhonghua 11 (ZH11) [5]. Meanwhile, the CaMV:: 35S-MTS-*orf116b*-GFP construct in the pPD1301 vector was inserted into the dicotyledon plants *Arabidopsis thaliana* following *Agrobacterium tumefaciens* (GV3101)-mediated infection of inflorescence [58] and tobacco following *Agrobacterium tumefaciens*-mediated infection of leaf explants [59]. Meanwhile, the MTS-*orf116b*-pCAMBIA2301 plant binary expression vector was inserted into Agrobacterium LBA4404, and these cells were infiltrated into the cotton cultivar R15 following the hypocotyl transformation method. The rice and cotton transgenic plants were maintained under field conditions, while the Arabidopsis and tobacco transgenic plants were maintained in a plant growth room at 21 °C and 25 °C, respectively. The leaves of all transgenic plants were collected at the seedling stage to extract DNA for PCR confirmation of the transgene, and the anthers were collected at the full bloom stage for cytological evaluation.

The anthers were collected from the transgenics between 8:00 A.M. and 10:00 A.M. and placed on two slides. The anthers on one slide were stained with 10 µL of 1% I_2_-KI and visualized and photographed using an ordinary optical microscope (Leica, Leica DM2500). Similarly, the anthers on the other slide were stained with 20 µL of the Alexander staining solution (Phygene Biotechnology Co., Ltd., Fuzhou, China), thoroughly mixed, covered with a cover glass, and incubated at room temperature. After 5–10 h, the anthers were visualized under an ordinary optical microscope, and the images were captured. The rice anthers and pollen grains were fixed in 2.5% glutaraldehyde for 24 h and visualized and photographed under an SEM to analyze pollen grain morphology (SU8100, Hitachi Limited Co., Ltd., Tokyo, Japan).

### 4.12. Construction of Split-Ubiquitin Membrane-Based Yeast Two-Hybrid (MYTH) System and Screening of Proteins Interacting with orf116b

A DUAL membrane library (www.dualsystems.com, accessed on 15 April 2023) [60], including membrane proteins, was constructed to screen the candidate proteins interacting with orf116b, which possesses a transmembrane structure (Figure S3A). Total RNA was extracted from the anthers of J4A and J4B at the full pollen stage, and the mRNA was purified using the oligo (dT) magnetic beads to construct a three-frame cDNA library. The double-stranded cDNA was generated from the purified mRNA and subjected to homologous recombination with linearized pPR3-N. Finally, the *E. coli* TOP10 cells were electro-transferred using the homologous recombination product. After confirming the library by PCR and electrophoresis, plasmids were extracted using the HighPure Maxi Plasmid Kit (Tiangen, DP116). Similarly, the *orf116b*-pBT3-STE bait plasmid was constructed and transformed into the NMY51 yeast strain, and these cells were allowed to grow on SD-Leu/His (SD-LH) plates with 3-amino-1,2,4-triazole (3AT) (0, 10, 20, 30, 40, 50, 75, and 100 mM) to detect self-activation. Further, the NMY51 yeast strain containing the *orf116b*-pBT3-STE bait plasmid was allowed to grow on SD-TLH and SD-TLHA plates, and the constructed cotton cDNA library was screened. The DNA extracted from the NMY51 positive clone (interaction) was sequenced, and the sequences were BLAST analyzed against the GenBank database. The gene encoding the interacting protein X was then inserted into the pPR3-N vector. Finally, to confirm the protein–protein interaction, the *orf116b*-pBT3-STE (bait) and the X-pPR3-N (prey) plasmids were co-transformed into yeast and screened on SD-Trp/Leu (SD-TL), SD-TLH, and SD-TLHA plates.

Further, the BiFC assay was carried out in tobacco plants to confirm the in vivo interaction between the candidate proteins using the *Agrobacterium tumefaciens* cells containing the pCAMBIA1300-N end of eYFP (YNE)-*orf116b*, pCAMBIA2300-C end of eYFP (YCE)-*LAT52*, YNE-*OsHAL3* (YN), YCE-*OsHAL3* (YC), YNE, or YCE constructs. Approximately 50 mL of the transformed bacterial solution (final concentration 10 mmol/L MgCl_2_, 10 mmol/L MES, 100 μmol/L AS) was filtered through a 0.1 μm filter and centrifuged at 5000 rpm for 10 min. The collected bacterial cell pellet was resuspended in the infection buffer to an OD_600_ of 1.0 and allowed to stand for 3 h at 25 °C. Then, the bacterial solutions with different constructs were then mixed in equal volumes [YNE-*orf116b* + YCE-*LAT52*, YN + YC (positive control), or YNE + YCE (negative control)] and injected at the back of the tobacco leaves with sufficient moisture (thoroughly watered for about 24 h). The co-transformed tobacco plants were maintained at 22 °C under weak light for 48 h, and the interaction was assessed by observing the leaves under a laser confocal microscope (STELLARIS 8, Wetzlar, Germany).

## 5. Conclusions

The present study identified the CMS-associated gene, *orf116b*, in the mt genome of the cotton CMS line J4A based on mt genome sequencing and comparison with its cytoplasmic nuclear homologous maintainer line J4B. Overexpression of *orf116b* in different plant species (rice, Arabidopsis, tobacco, and cotton) confirmed its role in determining CMS. Further analysis revealed that orf116b was toxic to *E. coli*, co-transcribed with *rpl2* and *rpl5* (the two functional genes encoding the ribosomal large subunits in mitochondria), and disrupted the mt function, indicating its role in regulating male sterility. We also found that ROS burst and ATP deficiency contributed to pollen abortion in J4A. In vitro analysis showed that orf116b probably interacts with LAT52, an anther-specific protein critical for microspore development. Thus, the study’s findings reveal the CMS mechanism and improve the potential of J4A in cotton hybrid seed production. Nevertheless, we will continue to delve deeper into the molecular mechanisms underlying the toxic effects of orf116b and its impact on the abnormal proliferation of tapetal cells.

## Data Availability

Data will be made available on request.

## Supplementary Materials

The following supporting information can be downloaded at: https://www.mdpi.com/article/10.3390/ijms252212257/s1.
