# Supplementary material for: Orf116b Induces Pollen Abortion in a Novel Cotton (Gossypium hirsutum L.) Cytoplasmic Male Sterile Line J4A
Source: Int J Mol Sci. 2024 Nov 14;25(22):12257. doi: 10.3390/ijms252212257 (PMC11594666; doi:10.3390/ijms252212257)
Supplement: Supplementary file 1 [file ijms-25-12257-s001.zip › Supplementary figures.pdf]

Supplementary figures

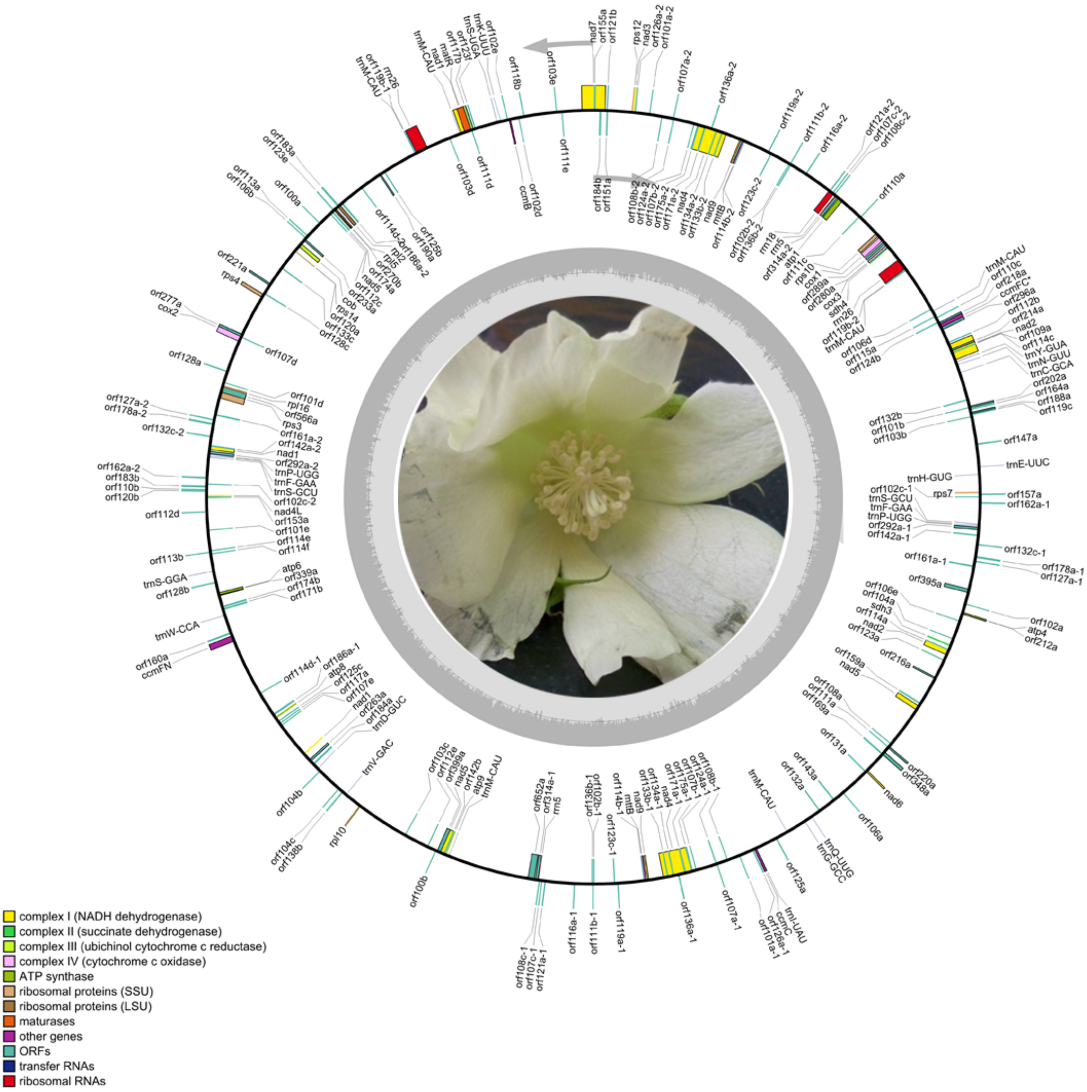

Figure S1. Circular map of the mt genome of the maintainer line J4B.

A B

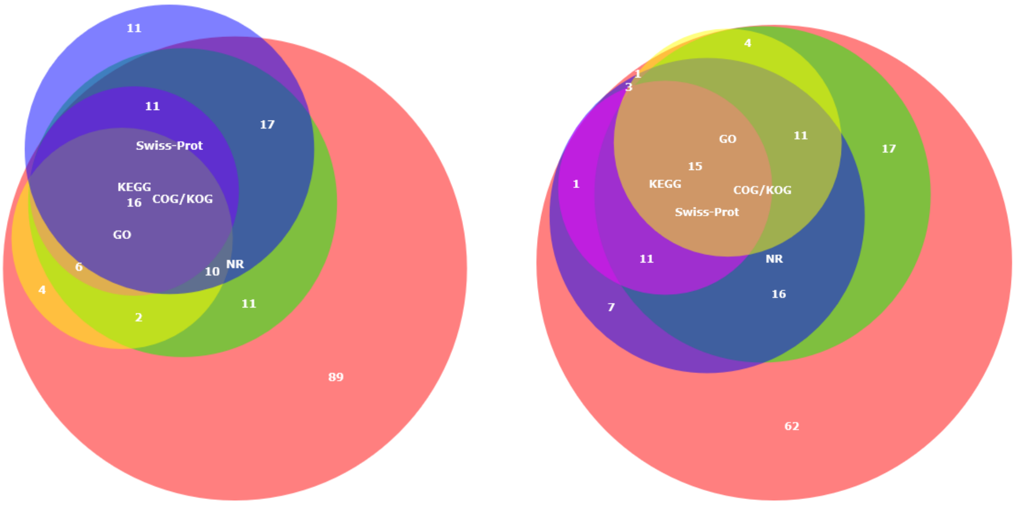

Figure S2. Annotations the mt genomes of the lines J4A and J4B based on the reference genome databases and classification according to the number of amino acids. Venn diagrams showed the number of genes annotated for the lines J4B (A) and J4A (B) based on NR, COG/KOG, GO, KEGG, and Swiss-Prot databases. Light brick red represents NR databases, green represents COG/KOG databases, blue purple represents Swiss Prot databases, yellow green represents GO databases, and purple represents KEGG databases.

A B

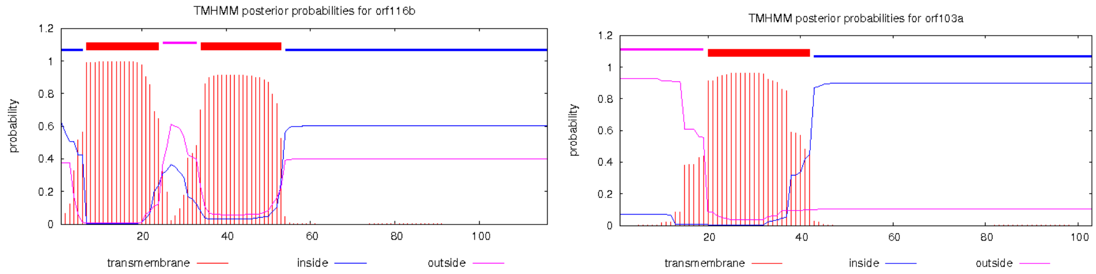

Figure S3. Prediction of transmembrane domains of *orf116b*.

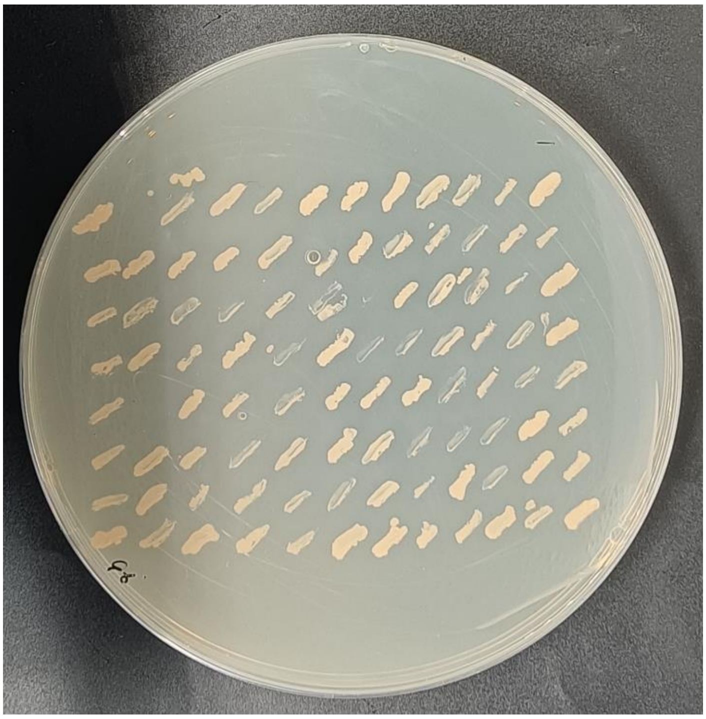

Figure S4. Ninety-six monoclonal colonies selected after preliminary screening.

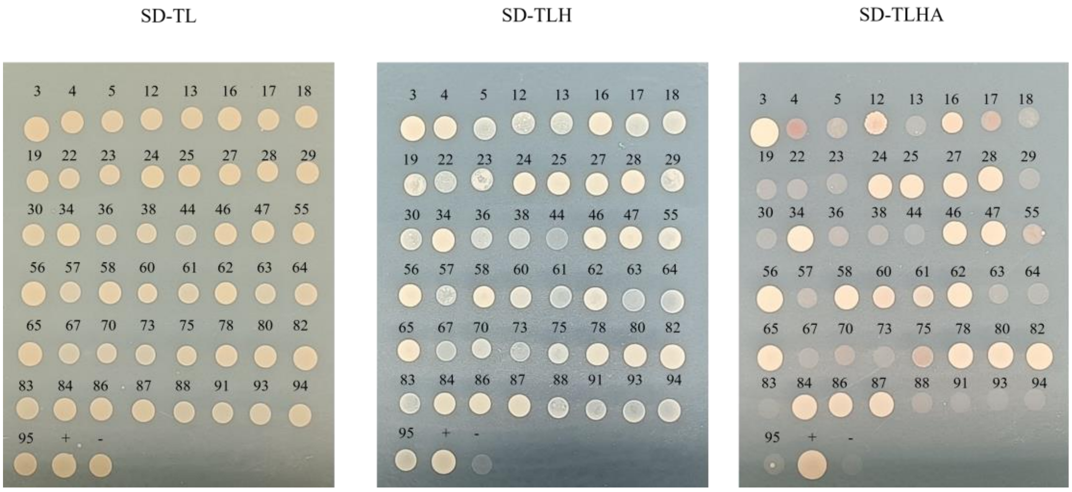

Figure S5. Validation of orf116b interacting protein based on a split-ubiquitin yeast two-hybrid system.

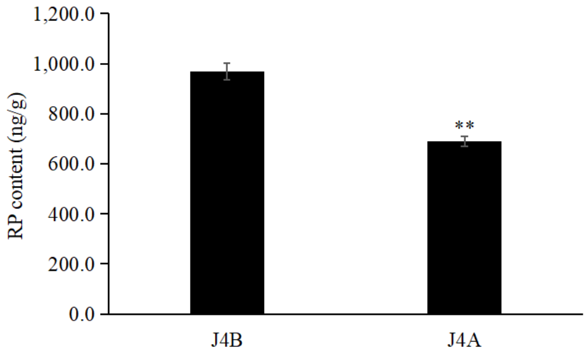

Figure S6. RP content of J4A and J4B.

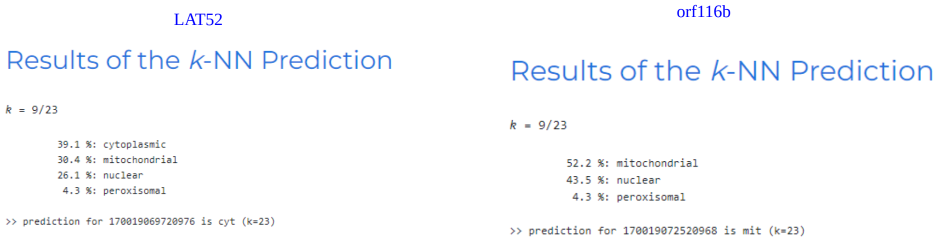

Figure S7. Subcellular localization prediction of LAT52 and orf116b (https://www.novopro.cn/tools/translate.html).

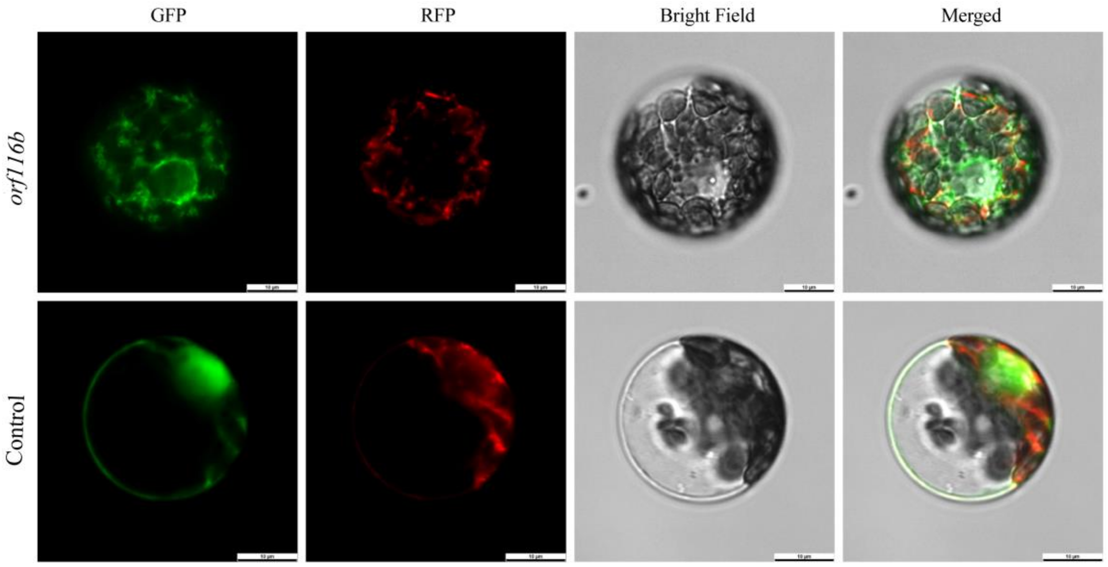

Figure S8. Subcellular localization of orf116b in Arabidopsis protoplasts. Green represents the signal from the green fluorescent protein (GFP); bright red indicates the mitochondrial localization signal; magenta indicates the autofluorescence of chloroplasts; orange-yellow and orange indicate the overlap of the green fluorescent

signal with the red fluorescent signal.
