# Supplementary material for: Orf116b Induces Pollen Abortion in a Novel Cotton (Gossypium hirsutum L.) Cytoplasmic Male Sterile Line J4A
Source: Int J Mol Sci. 2024 Nov 14;25(22):12257. doi: 10.3390/ijms252212257 (PMC11594666; doi:10.3390/ijms252212257)
Supplement: Supplementary file 1 [file ijms-25-12257-s001.zip › Supplementary tables.pdf]

Supplementary tables

Table S1. Statistics of mitochondrial sequencing.

**Sample ID**	**J4A**					
**Illumina PE**	**Insert size (nt)**	**Raw data (Mb)**	**Clean data (Mb)**	**Q20 (%)**	**Q30 (%)**	**GC (%)**
	450	11443.8	10991.4	95.22	86.55	39.29
PacBio	Read number	Read bases	Greatest length (nt)	N50 length (nt)	N90 length (nt)	Mean length (nt)
	92,527	460,703,329	209,202	5,245	3,654	4,979
**Sample ID**	**J4B**					
**Illumina PE**	**Insert size (nt)**	**Raw data (Mb)**	**Clean data (Mb)**	**Q20 (%)**	**Q30 (%)**	**GC (%)**
	450	8454.8	8147.3	95.37	86.95	38.99
PacBio	Read number	Read bases	Greatest length (nt)	N50 length (nt)	N90 length (nt)	Mean length (nt)
	117,674	574,874,134	194,861	5,132	3,611	4,885

Table S2. The characteristics of the assembled mt genome of J4A and J4B.

**Sample**	**Subreads Number**	**Subreads Reads Bases(bp)**	**Larget Length (bp)**	**N50 Length (bp)**	**N90 Length (bp)**	**Average Length (bp)**	**Total length (bp)**	**GC content (%)**	**N rate (%)**
J4A	92527	460703329	209202	5245	3654	4979	634005	44.91	0
J4B	117674	574874134	194861	5132	3611	4885	677303	44.96	0

Note：Samples: name of the sample; Subreads Number: the number of subreads of the sample after filtering; Subreads Bases (bp): the total amount of Ssubreads data ofin the sample after filtering; Larget Length (bp): the maximum length of the sample after filtering; N50 Length (bp): Subreads N50 length of the sample after filtering; N90 Length (bp): Subreads N90 length of the sample after filtering; Average Length (bp): the average subreads length after filtering. Total Length (bp): the

total length of the sample assembly sequence; GC Content (%): GC content of the sample assembly sequence; N rate (%): the content of unknown base N in the assembly sequence of the sample.

Table S3. Statistical table of gene function annotation results.

**Samples**	**Gene number**	**Gene total length(bp)**	**Gene average length(bp)**	**Gene length/Genome(%)**	**NR**	**GO**	**eggNOG**	**KEGG**	**Swiss**
J4A	193	104490	541	16.48	148	34	74	30	65
J4B	208	112824	542	16.66	167	38	81	34	74

Table S4. Genes contents of cotton mitotypes.

Product Group	Gene name	J4B	J4A
Complex I	*nad1*	+	+
	nad2	+	+
	nad3	+	+
	nad4	**+2[a]**	**+**
	nad4L	+	+
	nad5	+	+
	nad6	+	+
	nad7	+	+
	nad9	**+2[a]**	**+**
Complex II	*sdh3*	+	+
	sdh4	+	+
Complex III	*Cob*	+	+
Complex IV	*cox1*	+	+

	cox2	+	+
	cox3	+	+
	atp1	+	+
	atp4	+	+
Complex V	*atp6*	+	+
	atp8	+	+
	atp9	+	+
	ccmB	+	+
Cytochrome C	*ccmC*	+	+
	ccmFN	+	+
	ccmFC	+	+
Other gene	***mttB***	**+2[a]**	**+**
	matR	+	+
	rps3	+	+
	rps4	+	+
	rps7	+	+
	rps10	+	+
Ribosome	*rps12*	+	+
	rps14	+	+
	rpl2	+	+
	rpl5	+	+
	rpl10	+	+
	rpl16	+	+
Total protein-coding genes	**36**		
	trnW-CCA	+	+
	trnV-GAC	+	+
	trnD-GUC	**+**	**+2[a]**
	trnS-UGA	+	+

	trnS-GGA	+	+
	trnS-GCU	**+2[a]**	**+**
	trnF-GAA	**+2[a]**	**+**
	trnP-UGG	**+2[a]**	**+**
	trnY-GUA	+	+
	trnN-GUU	+	+
	trnC-GCA	+	+
	trnE-UUC	+	+
	trnH-GUG	+	+
	trnK-UUU	+	+
	trnQ-UUG	+	+
	trnG-GCC	+	+
	trnM-CAU	+6[a]	+5[a]
	trnI-UAU	+	+
Total tRNA genes		26	23
	rrn18	+	+2[a]
rRNA	*rrn5*	+2[a]	+2[a]
	rrn26	+2[a]	+2[a]
Total rRNA genes		5	6

+ denotes presence of multiple copies of the gene.

Table S5. The three classifications of ORFs in the mt genomes of J4A compared with J4B.

AS-ORFs	TM-ORFs	CH-ORFs
orf208a	*orf102b*	*orf212a*
orf116b	***orf103a***	*orf114a*
orf240a	*orf103d*	*orf131a*
orf103a	*orf108a*	*orf126a-2*
orf101c	*orf108b-1*	*orf175a*

orf109b	*orf108b-2*	*orf317a-2*
orf112a	*orf114a*	*orf228a*
orf123b	*orf114b*	*orf280a*
orf123d	*orf116a-2*	***orf116b***
orf133a	***orf116b***	*orf270b*
orf135a	*orf119b-1*	*orf123e*
orf138a-1	*orf119b-2*	*orf174a*
orf138a-2	*orf123f*	*orf120a*
orf150a	*orf128b*	*orf129b*
orf212b	*orf129a*	*orf277a*
	orf132a	*orf101d*
	orf132b	*orf566a*
	orf134a	*orf342a*
	orf138b	*orf592a*
	orf157a	*orf186a*
	orf159a	*orf126a-1*
	orf160a	*orf102e*
	orf162a	*orf111d*
	orf171a	*orf124b*
	orf175a	*orf218a*
	orf186a	
	orf202a	
	orf208a	
	orf214a	
	orf218a	
	orf240a	
	orf277a	
	orf280a	

orf296a
orf317a-1
orf317a-2
orf342a
orf399a
orf592a
orf610a

Table S6. Proteins that may interact with orf116b.

Number	Location	Annotation
>3	LOC107894327	Uncharacterized
**>12**	**LOC107931849**	**ribosome biogenesis protein NOP53**
>16	LOC107886462	REF/SRPP-like protein At1g67360
>24	LOC121229211	nucleolin-like
**>25**	**LOC107942900**	**anther-specific protein LAT52-like**
>27	LOC121225335	protein transport protein Sec61 subunit beta-like
>28	LOC107954684	GPI transamidase component PIG-T transcript variant X1
>34	LOC107916745	CTL-like protein DDB_G0288717
>46	LOC107906604	sugar transport protein 8-like
>47	LOC121217851	protein KRTCAP2 homolog
>56	LOC107901650	ER membrane protein complex subunit 4
>58	LOC107946532	protein Asterix
>60	LOC107899667	Uncharacterized
>61	LOC121216253	Uncharacterized
**>62**	**LOC107914072**	**NAC domain-containing protein 17 transcript variant X4**
>65	LOC107958994	Midasin
>78	LOC107923220	Uncharacterized
>80	LOC107904538	transmembrane protein 120 homolog transcript variant X1

>82	LOC121225673	reticulon-like protein B11
>84	LOC121202809	fasciclin-like arabinogalactan protein 1
>86	LOC107888331	Calreticulin
>87	LOC107943904	small ubiquitin-related modifier 1

Table S7. PCR primers used in this study.

Primer name	Primer sequence	Use
18S-F	ATCAGCTCGCGTTGACTACGT	
18S-R	ACACTTCACCGGACCATTCAAT	
qPCR_*orf116b*-F	CGGTCGCTAAAGAAAAGAGACTGGC	qPCR
qPCR_*orf116b*-R	GTAAGAGGAAGTACGAGCAGAAATC	
pGEX5'	GGGCTGGCAAGCCACGTTTGGTG	PGEX4T-1 vector primer
pGEX3'	CCGGGAGCTGCATGTGTCAGAGG	
PE6-F	gatctggttccgcgtggatccATGAATTGTCTAAGTGCTCTCCTCTCT	Construction of *orf116b*-pGEX4T-1 prokaryotic expression vector
PE6-R	ctcgagtcgacccgggaattcTCAGTCAATTCTTTCTTTTAGGAAGG	
PE3-F	gatctggttccgcgtggatccATGTTCCCTTACTCGGTTGTTCTT	Construction of *orf103a*-pGEX4T-1 prokaryotic expression vector
PE3-R	ctcgagtcgacccgggaattcCTAGCGCCTGTGGGTCCAC	
2O F	AGTAACTCTTCGCCTTTCAA	Co-transcriptional analysis of *orf116b-rpl2*
2O R	CACTACATAAGCCGCCATCA	
5O F	TCAAGATCCGTTGCTCAAAC	Co-transcriptional analysis of *orf116b-rpl5*
5O R	GACATAACATTCTCGGTCGC	

>orf116b

ATGAATTGTCTAAGTGCTCTCCTCTCTTGGCTTTGTCTCATTGTCTATCTCGTAATCATTCGAT
TCCGCGCTTCAATTGAAGCAAGCTCCTACTCCTCCTCCTTCTCCTTCGTATCCTTCATCGTCG
TTGGTCCTTTTGACATAACATTCTCGGTCGCTAAAGAAAAGAGACTGGCTCTCTCTCTATCT
ATTAAACGCAATATCTTGAAAGGCGAAGAGTTACTACTGATTTCTGCTCGTACTTCCTCTTAC
TGTAGTGATACAGTACTCGTGCAAGAGCGCTTACGGCAAGAGCTAATTCAGCTAAATCAATG
GCACGTGGGAGCCTTCCTAAAAGAAAGAATTGACTGA
